# Exploratory study on the self-perceived knowledge and care competence of general practitioners in managing patients with overweight and obesity in Austria

**DOI:** 10.1007/s00508-025-02545-3

**Published:** 2025-06-11

**Authors:** Sabine Fritzenwallner, Maria Flamm, Hans-Peter Wiesinger, Dagmar Schaffler-Schaden

**Affiliations:** 1 Master Programme Public Health, Center for Public Health and Healthcare Research, Paracelsus Medical University, Strubergasse 21, 5020 Salzburg, Austria; 2 Institute of General Practice, Family Medicine and Preventive Medicine, Center for Public Health and Healthcare Research, Paracelsus Medical University, Strubergasse 21, 5020 Salzburg, Austria; 3 Institute of Nursing Science and Practice, Center for Public Health and Healthcare Research, Paracelsus Medical University, Strubergasse 21, 5020 Salzburg, Austria

**Keywords:** Care competencies, Continuing education and training, Primary care, Quantitative survey/questionnaire, Barriers to care

## Abstract

**Objectives:**

Obesity is a chronic, multifactorial disease that is often described as an epidemic or pandemic of the 21st century. General practitioners (GPs) play a crucial role in managing it, yet multiple barriers hinder effective care. This study explores Austrian GPs’ experiences in obesity management, their self-assessed knowledge and care competencies, and the perceived barriers to optimal treatment.

**Methods:**

A quantitative online survey was conducted among GPs in October and November 2023. The questionnaire covered prevention, treatment, barriers, and experiences in managing overweight and obesity in primary care.

**Results:**

In all, 59 GPs (56% female) completed the survey. Nearly all recognized obesity as a chronic disease and felt responsible for its management, but almost half considered their training insufficient. While 80% acknowledged the need for multimodal therapy programs, awareness of available healthcare services was low. The most significant barrier to effective obesity care from the GP’s point of view was a lack of patient motivation. Female GPs were more likely to screen for dietary habits and physical activity, refer patients to specialized care, and request additional resources, whereas male GPs were more likely to prescribe pharmacotherapy. More experienced GPs felt better trained but were less likely to seek treatment guidelines. Regional and practice-setting differences influenced attitudes and referral patterns. GPs with private insurance contracts felt best trained and were least likely to request additional support.

**Conclusion:**

The survey revealed considerable uncertainty among GPs regarding the management of overweight and obesity. These findings highlight the need for targeted interventions to improve patient care and enhance GPs’ training in obesity management.

**Supplementary Information:**

The online version of this article (10.1007/s00508-025-02545-3) contains supplementary material, which is available to authorized users.

## Introduction

Obesity is a complex, multifactorial chronic disease, influenced by numerous endogenous and exogenous factors [1, 2]. The prevalence of overweight and obesity is increasing worldwide. In Austria, the prevalence of obesity is expected to increase annually by 2.4% among adults and 2.8% among children, with more than one-third of the adult population projected to be obese by 2035 [3]. Given these high numbers, primary care providers increasingly encounter overweight and obese patients. However, obesity is often underdiagnosed and undertreated, with primary care facing multiple barriers to providing adequate care [4–7].

In Austria, primary care is mainly delivered through solo practices, with 89% of contracted general practitioners (GPs) operating solo in 2018 [8]. The majority of primary care is provided by GPs with health insurance contracts, but there is a trend towards private practices [9, 10]. Regarding the treatment of chronic diseases, Austria has traditionally reported higher rates of potentially avoidable hospital admissions for chronic conditions compared to many European countries [10]. These rates have declined over the past decade and are now closer to the EU average. Despite this progress, Austria’s hospital-centered healthcare system continues to limit primary care’s ability to manage chronic diseases effectively. Efforts to strengthen primary care and reduce hospital admissions are ongoing, but structural and financial fragmentation remains challenging [10].

Currently, obesity treatment in primary care presents multiple challenges. Many guidelines highlight the importance of an interdisciplinary approach to care, but the structure of the primary care system limits access to these services [1, 11–13]. There are various therapy programs, mainly offered by health insurance funds in Austria’s federal states (e.g., “Easy Kids”, “Leichter Leben”), which mainly focus on the aspects of nutrition and exercise, but also on behavior. However, there is no standardized concept for structured obesity care in Austria. International comparisons, such as Germany’s recent steps toward a structured obesity disease management program (DMP) [14], highlight Austria’s need for improving coordination and resources.

The aim of this study was to explore GPs’ experiences in managing overweight and obesity in daily practice, self-assessed knowledge and care skills and barriers to optimal care in Austria.

## Materials and methods

### Study design and participants

A cross-sectional quantitative survey was conducted to assess the experiences of GPs in managing overweight and obesity in primary care and the barriers they face in their daily practice. Participants were included regardless of employment type, practice setting, or years of experience.

### Survey development and data collection

Since the survey was tailored to the regional characteristics of primary care in Austria, we developed a questionnaire. After an extensive literature search in PubMed and the identification of several studies with a similar research purpose [5, 15–17], we created a questionnaire, based on predefined points considered particularly relevant for general practice, as well as key aspects of prevention and treatment from clinical guidelines. Three GPs pretested the questionnaire for clarity and relevance. The survey consisted of 22 items, including 6 demographic items and 16 content-related questions about confidence in assessing obesity, managing obesity, barriers to optimal care, and knowledge about obesity prevention and treatment (Supplementary Tables 1–2). It was conducted using LimeSurvey.org©. The link to the survey, together with a brief description, was sent to family physicians in Vienna and Salzburg via the mailing lists of the Austrian Society of General Practice/Family Medicine (ÖGAM), the Salzburg (SAGAM) and Vienna (WIGAM) Society of General Practice/Family Medicine and the general medicine teaching physicians at the Paracelsus Medical University Salzburg. The survey was distributed from October 22 to November 7, 2023, with responses collected until November 13, 2023. All data were anonymized.

### Ethical considerations and data protection

Participants were informed about the project’s purpose and that their responses were confidential and would be used for research only. The participation was voluntary; responses were collected anonymously and could not be traced back to one of the participants. As no patients or vulnerable groups were involved and no health-related sensitive data were collected, ethical approval was not considered necessary for the survey.

### Data analysis and statistical methods

Descriptive statistics were presented as frequencies and percentages. The screening, prevention, and treatment questionnaires addressed conceptually distinct constructs—such as perceived responsibility, practitioners’ education, and specific interventions—that could not be meaningfully combined into a single model for regression analysis. Given these conceptual differences, the exploratory nature of the study, along with a small sample size and a skewed response distribution with several extreme categories lacking observations, regression analyses were considered methodologically inappropriate. Consequently, relationships between demographic characteristics and screening, prevention, and treatment aspects were analyzed using Pearson’s χ^2^ test or Fisher’s exact test for individual comparisons. Only fully completed questionnaires were included in the analysis, and no imputations for missing data were performed. All statistical analyses were conducted using SPSS Statistics 29.0 (IBM, Armonk, NY, USA).

## Results

Demographic characteristics of the GPs, along with their overall and subgroup-specific counts and percentages for each question related to screening, prevention, and treatment, are presented in Table 1 and contingency Tables 2, 3, 4 and 5. Of all the GPs contacted, 62 completed the online study, but only 59 did so entirely. These 59 GPs were included in the further evaluation.

**Table 1:** Demographic characteristics of the general practitioners

	Total *n *(%)
*Gender*	
Female	33 (56)
Male	26 (44)
*Experience as a general practitioner*	
< 5 years	19 (32)
5–10 years	17 (29)
11–20 years	14 (24)
> 20 years	9 (15)
*Chronological age of general practitioners*	
30–39	15 (25)
40–49	24 (41)
50–59	13 (22)
60–69	6 (10)
> 70	1 (2)
*Federal state*	
Vienna	17 (29)
Salzburg	34 (58)
Other	8 (13)
*Setting of general practice*	
Solo practice	35 (59)
Group practice	8 (14)
Primary care unit	14 (24)
Other	2 (3)
*Health insurance contract*	
Yes, with all insurances	44 (75)
No, working as a private practitioner	2 (3)
No, employed or working as a substitute doctor	13 (22)

**Table 2:** Screening parameters and prevention measures of GPs in relation to gender, experience and age

	Total	Gender		Experience (years)				Age (years)				
		♀	♂	< 5	5–10	11–20	> 20	30–39	40–49	50–59	60–69	≥ 70
**Doctors task**												
Very strongly	24(41)	*16(49)*	*8(31)*	*5(26)*	*10(59)*	*4(29)*	*5(56)*	*3(20)*	*11(46)*	*6(46)*	*3(50)*	*1(100)*
Strongly*	28(48)	*14(42)*	*14(54)*	*13(68)*	*5(29)*	*6(43)*	*4(44)*	*9(60)*	*10(42)*	*6(46)*	*3(50)*	*0(0)*
Barely	7(12)	*3(9)*	*4(15)*	*1(5)*	*2(12)*	*4(29)*	*0(0)*	*3(20)*	*3(13)*	*1(8)*	*0(0)*	*0(0)*
**Screening parameters**^**a**^												
BMI*	57(97)	*31(94)*	*26(100)*	*19(100)*	*16(94)*	*14(100)*	*8(89)*	*14(93)*	*24(100)*	*12(92)*	*6(100)*	*1(100)*
Waist-to-hip ratio	10(17)	*7(21)*	*3(12)*	*3(16)*	*3(18)*	*2(14)*	*2(22)*	*3(20)*	*2(8)*	*4(31)*	*0(0)*	*1(100)*
Waist circumference	35(59)	21(64)	14(54)	9(47)	13(77)	9(64)	4(44)	*6(40)*	*16(67)*	*10(77)*	*2(33)*	*1(100)*
Family history	35(59)	21(64)	14(54)	10(53)	10(59)	9(64)	6(67)	*8(53)*	*14(58)*	*9(70)*	*4(67)*	*0(0)*
Dietary habits	44(75)	**28(85)**	**16(62)**	*14(74)*	*13(77)*	*11(79)*	*6(67)*	*11(73)*	*20(83)*	*9(69)*	*3(50)*	*1(100)*
Physical activity	47(80)	**30(91)**	**17(65)**	***15(79)***	***17(100)***	***9(64)***	***6(67)***	*12(80)*	*21(88)*	*9(69)*	*4(67)*	*1(100)*
Blood tests	49(83)	*28(85)*	*21(81)*	*17(90)*	*14(82)*	*11(79)*	*7(78)*	*13(87)*	*20(83)*	*11(85)*	*4(67)*	*1(100)*
Other	3(5)	*2(6)*	*1(4)*	*1(5)*	*1(6)*	*0(0)*	*1(11)*	*1(7)*	*1(4)*	*1(7)*	*0(0)*	*0(0)*
**Prevention measures**^**a**^												
Nutrition rec.	55(93)	*31(94)*	*24(92)*	*17(90)*	*15(88)*	*14(100)*	*9(100)*	*13(87)*	*22(92)*	*13(100)*	*6(100)*	*1(100)*
Physical activity rec.*	59(100)	33(100)	26(100)	19(100)	17(100)	14(100)	9(100)	*15(100)*	*24(100)*	*13(100)*	*6(100)*	*1(100)*
Behavioral rec.	30(51)	20(61)	10(39)	*11(58)*	*9(53)*	*7(50)*	*3(33)*	*9(60)*	*13(54)*	*6(46)*	*2(33)*	*0(0)*
Weight monitoring	35(59)	21(64)	14(54)	10(53)	12(71)	6(43)	7(78)	*6(40)*	*15(63)*	*7(54)*	*6(100)*	*1(100)*
Pharmacological therapy	10(17)	*4(12)*	*6(23)*	*1(5)*	*3(18)*	*3(21)*	*3(33)*	*2(13)*	*4(17)*	*2(15)*	*1(17)*	*1(100)*
Referral to specialists	29(49)	19(58)	10(39)	*11(58)*	*11(65)*	*4(29)*	*3(33)*	*9(60)*	*14(58)*	*5(39)*	*1(17)*	*0(0)*
Other	2(3)	*2(6)*	*0(0)*	*0(0)*	*2(12)*	*0(0)*	*0(0)*	*1(7)*	*1(4)*	*0(0)*	*0(0)*	*0(0)*

Counts and percentages in **bold **show a significant effect of the demographic variable, and *Fisher’s exact test*—expected frequencies too low—was used for those in *italics*

*rec.* recommendations, *BMI* body mass index

^a^Only counts and percentages of positive confirmations are provided

*Shows the highest overall response

**Table 3:** Screening parameters and prevention measures of GPs in relation to federal state, practice setting and insurance contract

	Federal state				Setting				Insurance		
	Total	Vienna	Sbg.	Other	Solo	Group	PCU	Other	Yes (all)	No, priv.	No, s./e.
**Doctors task**											
Very strongly	24(41)	*6(35)*	*15(44)*	*3(38)*	*15(43)*	*5(63)*	*3(21)*	*1(50)*	*18(41)*	*1(50)*	*5(39)*
Strongly	28(48)	*9(53)*	*14(41)*	*5(63)*	*16(46)*	*2(25)*	*9(64)*	*1(50)*	*20(46)*	*0(0)*	*8(62)*
Barely	7(12)	*2(12)*	*5(15)*	*0(0)*	*4(11)*	*1(13)*	*2(14)*	*0(0)*	*6(14)*	*1(50)*	*0(0)*
**Screening parameters**^**a**^											
BMI	57(97)	*17(100)*	*33(97)*	*7(88)*	*34(97)*	*7(88)*	*14(100)*	*2(100)*	*42(96)*	*2(100)*	*13(100)*
Waist-to-hip ratio	10(17)	*3(18)*	*5(15)*	*2(25)*	*8(23)*	*1(13)*	*1(7)*	*0(0)*	*9(21)*	*1(50)*	*0(0)*
Waist circumference	35(59)	*9(53)*	*19(56)*	*7(88)*	*25(71)*	*4(50)*	*6(43)*	*0(0)*	*27(61)*	*2(100)*	*6(46)*
Family history	35(59)	*9(53)*	*20(59)*	*6(75)*	*22(63)*	*5(63)*	*6(43)*	*2(100)*	*27(61)*	*1(50)*	*7(54)*
Dietary habits	44(75)	*12(71)*	*27(80)*	*5(63)*	*28(80)*	*7(88)*	*7(50)*	*2(100)*	*32(73)*	*2(100)*	*10(77)*
Physical activity	47(80)	*16(94)*	*26(77)*	*5(63)*	*27(77)*	*8(100)*	*10(71)*	*2(100)*	*33(75)*	*2(100)*	*12(92)*
Blood tests	49(83)	*14(82)*	*29(85)*	*6(75)*	*29(83)*	*7(88)*	*11(79)*	*2(100)*	*37(84)*	*2(100)*	*10(77)*
Other	3(5)	*1(6)*	*1(3)*	*1(13)*	2(6)	*0(0)*	*1(7)*	*0(0)*	*2(5)*	*0(0)*	*1(8)*
**Prevention measures**^**a**^											
Nutrition rec.	55(93)	*16(94)*	*33(97)*	*6(75)*	*33(94)*	*7(88)*	*13(93)*	*2(100)*	*41(93)*	*2(100)*	*12(92)*
Physical activity rec.	59(100)	*17(100)*	*34(100)*	*8(100)*	*35(100)*	*8(100)*	*14(100)*	*2(100)*	*44(100)*	*2(100)*	*13(100)*
Behavioral rec.	30(51)	*8(47)*	*19(56)*	*3(38)*	*18(51)*	*4(50)*	*6(43)*	*2(100)*	*22(50)*	*1(50)*	*7(54)*
Weight monitoring	35(59)	*11(65)*	*21(62)*	*3(38)*	*22(63)*	*4(50)*	*8(57)*	*1(50)*	*26(59)*	*2(100)*	*7(54)*
Pharmacological therapy	10(17)	*4(24)*	*3(9)*	*3(38)*	*7(20)*	*1(13)*	*2(14)*	*0(0)*	*8(18)*	*1(50)*	*1(8)*
Referral to specialists	29(49)	*9(53)*	*15(44)*	*5(63)*	*17(49)*	*4(50)*	*6(43)*	*2(100)*	*20(46)*	*0(0)*	*9(69)*
Other	2(3)	*0(0)*	*2(6)*	*0(0)*	*1(3)*	*1(13)*	*0(0)*	*0(0)*	*1(2)*	*0(0)*	*1(8)*

Counts and percentages in **bold **show a significant effect of the demographic variable, and *Fisher’s exact test*—expected frequencies too low—was used for those in *italics*.

*Priv.* private, *s./e.* substitute/employee, *rec.* recommendations, *BMI* body mass index, *Sbg.* Salzburg

^a^Only counts and percentages of positive confirmations are provided

**Table 4:** Obesity treatment measures in relation to gender, experience and age

	Total	Gender		Experience (years)				Age (years)				
		♀	♂	< 5	5–10	11–20	> 20	30–39	40–49	50–59	60–69	≥ 70
**Chronic disease**												
Very strongly*	41(70)	*26(79)*	*15(58)*	*12(63)*	*14(82)*	*10(71)*	*5(56)*	*10(67)*	*18(75)*	*9(69)*	*4(67)*	*0(0)*
Strongly	15(25)	*7(21)*	*8(31)*	*5(26)*	*3(18)*	*4(29)*	*3(33)*	*4(26)*	*5(21)*	*4(31)*	*1(17)*	*1(100)*
Barely	3(5)	*0(0)*	*3(12)*	*2(11)*	*0(0)*	*0(0)*	*1(11)*	*1(7)*	*1(4)*	*0(0)*	*1(17)*	*0(0)*
**Doctors task**												
Very strongly	20(34)	*9(27)*	*11(42)*	***3(16)***	***6(35)***	***4(29)***	***7(78)***	*5(33)*	*4(17)*	*5(39)*	*5(83)*	*1(100)*
Strongly*	34(58)	*20(61)*	*14(54)*	***14(74)***	***11(65)***	***7(50)***	***2(22)***	*9(60)*	*17(71)*	*7(54)*	*1(17)*	*0(0)*
Barely	5(9)	*4(12)*	*1(4)*	***2(11)***	***0(0)***	***3(21)***	***0(0)***	*1(7)*	*3(13)*	*1(8)*	*0(0)*	*0(0)*
**Sufficiently trained**												
Very strongly	8(14)	*4(12)*	*4(15)*	***0(0)***	***3(18)***	***1(7)***	***4(44)***	***0(0)***	***4(17)***	***1(8)***	***2(33)***	***1(100)***
Strongly	23(39)	*10(30)*	*13(50)*	***6(32)***	***6(35)***	***6(43)***	***5(56)***	***6(40)***	***6(25)***	***7(54)***	***4(67)***	***0(0)***
Barely*	28(48)	*19(58)*	*9(35)*	***13(68)***	***8(47)***	***7(50)***	***0(0)***	***9(60)***	***14(58)***	***5(39)***	***0(0)***	***0(0)***
**Primary physician responsibility**												
Very strongly	17(29)	*9(27)*	*8(31)*	*3(16)*	*3(18)*	*5(36)*	*6(67)*	*4(27)*	*4(17)*	*5(39)*	*4(67)*	*0(0)*
Strongly*	37(63)	*20(61)*	*17(65)*	*14(74)*	*12(71)*	*8(57)*	*3(33)*	*9(60)*	*19(79)*	*6(46)*	*2(33)*	*1(100)*
Barely	5(9)	*4(12)*	*1(4)*	*2(11)*	*2(12)*	*1(7)*	*0(0)*	*2(13)*	*1(4)*	*2(15)*	*0(0)*	*0(0)*
**Treatment measures**^**a**^												
Nutrition rec.*	59(100)	*33(100)*	*26(100)*	19(100)	17(100)	14(100)	9(100)	15(100)	24(100)	13(100)	6(100)	1(100)
Physical activity rec.	56(95)	*30(91)*	*26(100)*	***19(100)***	***17(100)***	***11(79)***	***9(100)***	*15(100)*	*23(96)*	*11(85)*	*6(100)*	*1(100)*
Behavioral rec.	32(54)	19(58)	13(50)	*11(58)*	*10(59)*	*8(57)*	*3(33)*	*8(53)*	*16(67)*	*6(46)*	*2(33)*	*0(0)*
Pharmacological therapy	28(48)	**10(30)**	**18(69)**	*9(47)*	*9(53)*	*5(36)*	*5(56)*	*7(47)*	*14(58)*	*3(23)*	*3(50)*	*1(100)*
Other	9(15)	*6(18)*	*3(12)*	*1(5)*	*4(24)*	*1(7)*	*3(33)*	*3(20)*	*2(8)*	*2(15)*	*2(33)*	*0(0)*
**Referral to**^**a**^												
Specialist outpatient care	25(42)	**18(55)**	**7(27)**	9(47)	9(53)	4(29)	3(33)	*7(47)*	*13(54)*	*4(31)*	*1(17)*	*0(0)*
Dietitian/Nutritionist*	50(85)	*28(85)*	*22(85)*	*18(95)*	*15(88)*	*10(71)*	*7(78)*	*14(93)*	*22(92)*	*8(62)*	*5(83)*	*1(100)*
Physio‑/Exercise therapy	23(39)	13(40)	10(39)	8(42)	7(41)	5(36)	3(33)	*3(20)*	*13(54)*	*5(39)*	*1(17)*	*1(100)*
Psychotherapist/-logist	27(46)	17(52)	10(39)	*6(32)*	*9(53)*	*5(36)*	*7(78)*	*5(33)*	*11(46)*	*5(39)*	*5(83)*	*1(100)*
Obesity clinic/center	45(76)	27(82)	18(70)	*17(90)*	*14(82)*	*8(57)*	*6(67)*	*13(87)*	*20(83)*	*8(62)*	*4(67)*	*0(0)*
None	1(2)	*0(0)*	*1(4)*	*0(0)*	*0(0)*	*1(7)*	*0(0)*	*0(0)*	*0(0)*	*1(8)*	*0(0)*	*0(0)*
Other	5(9)	*3(9)*	*2(8)*	*1(5)*	*4(24)*	*0(0)*	*0(0)*	*4(27)*	*1(4)*	*0(0)*	*0(0)*	*0(0)*
**Conduction of long-term treatment**												
Very many	6(10)	*3(9)*	*3(12)*	*1(5)*	*1(6)*	*2(14)*	*2(22)*	*0(0)*	*2(8)*	*3(23)*	*1(17)*	*0(0)*
Many	18(31)	*9(27)*	*9(35)*	*4(21)*	*3(18)*	*6(43)*	*5(56)*	*1(7)*	*8(33)*	*5(39)*	*3(50)*	*1(100)*
Few*	23(39)	*12(36)*	*11(42)*	*8(42)*	*10(59)*	*4(29)*	*1(11)*	*7(47)*	*11(46)*	*4(31)*	*1(17)*	*0(0)*
No	12(20)	*9(27)*	*3(12)*	*6(32)*	*3(18)*	*2(14)*	*1(11)*	*7(47)*	*3(13)*	*1(8)*	*1(17)*	*0(0)*
**Familiarity with multimodal therapy programs**^**a**^												
Yes	33(56)	21(64)	12(46)	12(63)	9(53)	6(43)	6(67)	*11(73)*	*11(46)*	*6(46)*	*5(83)*	*0(0)*
**Barriers experienced**^**a**^												
Lack of motivation*	48(81)	*27(82)*	*21(81)*	*14(74)*	*16(94)*	*11(79)*	*7(78)*	*14(93)*	*18(75)*	*10(77)*	*5(83)*	*1(100)*
Lack of time	42(71)	24(73)	18(69)	*16(84)*	*12(71)*	*9(64)*	*5(56)*	*13(87)*	*17(71)*	*7(54)*	*4(67)*	*1(100)*
Limited knowledge	17(29)	10(30)	7(27)	*10(53)*	*4(24)*	*2(14)*	*1(11)*	*6(40)*	*8(33)*	*2(15)*	*1(17)*	*0(0)*
Lack of reimbursement	44(75)	26(79)	18(69)	*15(79)*	*13(77)*	*11(79)*	*5(56)*	*13(87)*	*17(71)*	*10(77)*	*3(50)*	*1(100)*
Costs for patients	42(71)	25(76)	17(65)	*16(84)*	*12(71)*	*9(64)*	*5(56)*	*13(87)*	*15(63)*	*11(85)*	*2(33)*	*1(100)*
Other	11(19)	*6(18)*	*5(19)*	*2(11)*	*3(18)*	*3(21)*	*3(33)*	*3(20)*	*3(13)*	*3(23)*	*2(33)*	*0(0)*
**Support desired**^**a**^												
Training programs	31(53)	18(55)	13(50)	*13(69)*	*10(59)*	*6(43)*	*2(22)*	*10(67)*	*13(54)*	*6(46)*	*2(33)*	*0(0)*
More spec. resources*	48(81)	***30(91)***	***18(69)***	*16(84)*	*15(88)*	*10(71)*	*7(78)*	*13(87)*	*21(88)*	*9(69)*	*4(67)*	*1(100)*
Interdisciplinary collab.	38(64)	21(64)	17(65)	*15(79)*	*11(65)*	*7(50)*	*5(56)*	*11(73)*	*18(75)*	*5(39)*	*4(67)*	*0(0)*
Guidelines/rec.	33(56)	21(64)	12(46)	**16(84)**	**10(65)**	**5(36)**	**2(11)**	***12(80)***	***14(58)***	***6(46)***	***1(17)***	***0(0)***
DMP	41(70)	21(64)	20(77)	*17(90)*	*11(65)*	*8(57)*	*5(56)*	*14(93)*	*15(63)*	*7(54)*	*4(67)*	*1(100)*
Other	5(9)	*3(9)*	*2(8)*	*2(11)*	*2(12)*	*1(7)*	*0(0)*	*3(20)*	*1(4)*	*1(8)*	*0(0)*	*0(0)*

Counts and percentages in **bold **show a significant effect of the demographic variable, and *Fisher’s exact test*—expected frequencies too low—was used for those in *italics*

*Rec.* recommendations, *spec.* specialized, *collab.* collaboration, *DMP* Disease Management Programme

^a^Only counts and percentages of positive confirmations are provided

*Shows the highest overall response

**Table 5:** Obesity treatment measures in relation to federal state, setting and insurance contract

	Total	Federal state		Setting					Insurance			
		Vienna	Sbg.	Other		Solo	Group	PCU	Other	Yes (all)	No, priv.	No, s./e.
**Chronic disease**												
Very strongly	41(70)	***8(47)***	***28(82)***	***5(63)***	***27(77)***		***8(100)***	***4(29)***	***2(100)***	*32(73)*	*1(50)*	*8(62)*
Strongly	15(25)	***8(47)***	***4(12)***	***3(38)***	***7(20)***		***0(0)***	***8(57)***	***0(0)***	*9(21)*	*1(50)*	*5(39)*
Barely	3(5)	***1(6)***	***2(6)***	***0(0)***	***1(3)***		***0(0)***	***2(14)***	***0(0)***	*3(7)*	*0(0)*	*0(0)*
**Doctors task**												
Very strongly	20(34)	*4(24)*	*12(35)*	*4(50)*	*14(40)*		*3(38)*	*3(21)*	*0(0)*	*16(36)*	*1(50)*	*3(23)*
Strongly	34(58)	*13(77)*	*17(50)*	*4(50)*	*17(49)*		*4(50)*	*11(79)*	*2(100)*	*23(52)*	*1(50)*	*10(77)*
Barely	5(9)	*0(0)*	*5(15)*	*0(0)*	*4(11)*		*1(13)*	*0(0)*	*0(0)*	*5(11)*	*0(0)*	*0(0)*
**Sufficiently trained**												
Very strongly	8(14)	*1(6)*	*4(12)*	*3(38)*	*7(20)*		*1(13)*	*0(0)*	*0(0)*	***5(11)***	***2(100)***	***1(8)***
Strongly	23(39)	*4(24)*	*17(50)*	*2(25)*	*14(40)*		*3(38)*	*6(43)*	*0(0)*	***20(46)***	***0(0)***	***3(23)***
Barely	28(48)	*12(71)*	*13(38)*	*3(38)*	*14(40)*		*4(50)*	*8(57)*	*2(100)*	***19(43)***	***0(0)***	***9(69)***
**Primary Physician responsibility**												
Very strongly	17(29)	*4(24)*	*11(32)*	*2(25)*	*11(31)*		*4(50)*	*1(7)*	*1(50)*	*12(27)*	*1(50)*	*4(31)*
Strongly	37(63)	*11(65)*	*20(59)*	*6(75)*	*21(60)*		*3(38)*	*12(86)*	*1(50)*	*27(61)*	*1(50)*	*9(69)*
Barely	5(9)	*2(12)*	*3(9)*	*0(0)*	*3(9)*		*1(13)*	*1(7)*	*0(0)*	*5(11)*	*0(0)*	*0(0)*
**Treatment measures**^**a**^												
Nutrition rec.	59(100)	17(100)	34(100)	8(100)	35(100)		8(100)	14(100)	2(100)	44(100)	2(100)	13(100)
Physical activity rec.	56(95)	*16(94)*	*33(97)*	*7(88)*	*32(91)*		*8(100)*	*14(100)*	*2(100)*	*41(93)*	*2(100)*	*13(100)*
Behavioral rec.	32(54)	*9(53)*	*19(56)*	*4(50)*	*19(54)*		*4(50)*	*7(50)*	*2(100)*	*22(50)*	*1(50)*	*32(54)*
Pharmacological therapy	28(48)	*8(47)*	*16(47)*	*4(50)*	*17(49)*		*3(38)*	*8(57)*	*0(0)*	*19(43)*	*2(100)*	*7(54)*
Other	9(15)	*1(6)*	*7(21)*	*1(13)*	*6(17)*		*3(38)*	*0(0)*	*0(0)*	*6(14)*	*0(0)*	*3(23)*
**Referral to**^**a**^												
Specialist outpatient care	25(42)	*6(35)*	*17(50)*	*2(25)*		*16(46)*	*4(50)*	*3(21)*	*2(100)*	*19(43)*	*1(50)*	*5(39)*
Dietitian/Nutritionist	50(85)	*15(88)*	*28(82)*	*7(88)*		*28(80)*	*7(88)*	*13(93)*	*2(100)*	*36(82)*	*2(100)*	*12(92)*
Physio‑/Exercise ther.	23(39)	*6(35)*	*13(38)*	*4(50)*		*15(43)*	*2(25)*	*5(36)*	*1(50)*	*16(36)*	*2(100)*	*5(39)*
Psychother./Psychologists	27(46)	*5(29)*	*18(53)*	*4(50)*		***18(51)***	***6(75)***	***2(14)***	***1(50)***	*22(50)*	*1(50)*	*4(31)*
Obesity clinic/center	45(76)	*15(88)*	*26(77)*	*4(50)*		*23(66)*	*7(88)*	*13(93)*	*2(100)*	***33(75)***	***0(0)***	***12(92)***
None	1(2)	*0(0)*	*1(3)*	*0(0)*		*1(3)*	*0(0)*	*0(0)*	*0(0)*	*1(2)*	*0(0)*	*0(0)*
Other	5(9)	*1(6)*	*3(9)*	*1(13)*		*2(6)*	*2(25)*	*1(7)*	*0(0)*	*3(7)*	*0(0)*	*2(15)*
**Conduction of long-term treatment**												
Very many	6(10)	*1(6)*	*5(15)*	*0(0)*		*6(17)*	*0(0)*	*0(0)*	*0(0)*	***6(14)***	***0(0)***	***0(0)***
Many	18(31)	*4(24)*	*10(29)*	*4(50)*		*10(29)*	*4(50)*	*4(29)*	*0(0)*	***15(34)***	***2(100)***	***1(8)***
Few	23(39)	*7(41)*	*12(35)*	*4(50)*		*13(37)*	*3(38)*	*6(43)*	*1(50)*	***17(39)***	***0(0)***	***6(46)***
No	12(20)	*5(29)*	*7(21)*	*0(0)*		*6(17)*	*1(13)*	*4(29)*	*1(50)*	***6(14)***	***0(0)***	***6(46)***
**Familiar with multimodal therapy programs**^**a**^												
Yes	33(56)	***6(35)***	***25(74)***	***2(25)***	*20(57)*		*7(88)*	*5(36)*	*1(50)*	*26(59)*	*0(0)*	*7(54)*
**Barriers experienced**^**a**^												
Lack of motivation	48(81)	*12(71)*	*28(82)*	*8(100)*		*28(80)*	*7(88)*	*11(79)*	*2(100)*	*36(82)*	*2(100)*	*10(77)*
Lack of time	42(71)	*13(77)*	*25(74)*	*4(50)*		*22(63)*	*7(88)*	*11(79)*	*2(100)*	*29(66)*	*2(100)*	*11(85)*
Limited knowledge	17(29)	*8(47)*	*7(21)*	*2(25)*		***5(14)***	***3(38)***	***7(50)***	***2(100)***	*11(25)*	*0(0)*	*6(46)*
Lack of reimbursement	44(75)	*14(82)*	*26(77)*	*4(50)*		*28(80)*	*5(63)*	*9(64)*	*2(100)*	*34(77)*	*2(100)*	*8(62)*
Costs for patients	42(71)	*13(77)*	*24(71)*	*5(63)*		*26(74)*	*4(50)*	*10(71)*	*2(100)*	*29(66)*	*2(100)*	*11(85)*
Other	11(19)	*1(6)*	*10(29)*	*0(0)*		*8(23)*	*1(13)*	*1(7)*	*1(50)*	*6(14)*	*1(50)*	*4(31)*
**Support desired**^**a**^												
Training programs	31(53)	*10(59)*	*19(56)*	*2(25)*		*16(46)*	*3(38)*	*10(71)*	*2(100)*	*20(46)*	*1(50)*	*10(77)*
Specialized resources	48(81)	*14(82)*	*27(79)*	*7(88)*		*28(80)*	*8(100)*	*10(71)*	*2(100)*	*35(80)*	*2(100)*	*11(85)*
Interdisciplinary collab.	38(64)	13(77)	21(62)	4(50)		*19(54)*	*6(75)*	*11(79)*	*2(100)*	*26(59)*	*1(50)*	*11(85)*
Guidelines/rec.	33(56)	*11(65)*	*19(56)*	*3(38)*		*16(46)*	*5(63)*	*10(71)*	*2(100)*	*23(52)*	*0(0)*	*10(77)*
DMP	41(70)	14(82)	23(68)	4(50)		***20(57)***	***5(63)***	***14(100)***	***2(100)***	*27(61)*	*2(100)*	*12(92)*
Other	5(9)	*0(0)*	*5(15)*	*0(0)*		*3(9)*	*1(13)*	*0(0)*	*1(50)*	***1(2)***	***0(0)***	***4(31)***

Counts and percentages in **bold **show a significant effect of the demographic variable, and *Fisher’s exact test*—expected frequencies too low—was used for those in *italics*

*Priv.* private, *s./e.* substitute/employee, *rec.* recommendations, *ther.* therapist

^a^Only counts and percentages of positive confirmations are provided

Nearly all GPs considered obesity a chronic disease and viewed themselves as the primary contact for its management. Consequently, all GPs felt responsible for preventing or treating overweight and obesity, and all used multiple parameters for screening, prevention and treatment. However, nearly half perceived their education as insufficient to adequately manage obesity, and almost all routinely referred their patients to other healthcare professionals. Approximately 80% of GPs recognized the need for multimodal therapy programs, though awareness of existing healthcare services for obesity management was generally low. From the GP’s perspective, the most significant barrier to effective obesity care was a lack of patient motivation. Each demographic factor, such as gender, professional experience, age, federal state, practice setting, and type of insurance contract, showed a significant association with at least one of the 16 content-related items on screening, prevention, or treatment measures.

Female GPs were approximately three and five times more likely to screen for dietary habits (χ^2^(1) = 4.17, OR = 3.50, 95% CI: 1.01–12.01, *p* = 0.041) and physical activity (χ^2^(1) = 5.85, OR = 5.29, 95%CI: 1.26–22.22, *p* = 0.016), respectively, than their male counterparts (Table 2). Additionally, they have shown higher odds of referring overweight and obese patients to specialized outpatient care (χ^2^(1) = 4.54, OR = 3.26, 95%CI: 1.08–9.82, *p* = 0.033), and expressed a greater demand for more specific resources to support their patients (χ^2^(1) = 4.51, OR = 4.44, 95%CI: 1.04–19.02, *p* = 0.034). In contrast, male GPs were over five times more likely to prescribe pharmacotherapy (χ^2^(1) = 8.84, OR = 5.29, 95%CI 1.69–16.67, *p* = 0.003, Table 4).

Fisher’s exact test revealed that all other demographic measures (years of experience, chronological age, federal state, practice setting, and insurance status of GPs) showed a significant association with at least one screening or treatment measure. Counts and percentages indicated that more experienced GPs seem more likely to screen for physical activity in obesity prevention (*p* = 0.029, Table 2), perceived a stronger responsibility for treating obesity (*p* = 0.019), felt better trained (*p* = 0.005), and were less likely to seek guidelines for patient support (*p* < 0.001). Years of experience were also associated with physical activity recommendations as a treatment, with GPs in the 11–20 years’ experience group appearing to recommend it less frequently (Table 4). Chronological age was significantly associated with feeling adequately trained (*p* = 0.017) and seeking treatment guidelines (*p* = 0.007). None of the GPs aged 60 or older felt inadequately trained, and only about 1 in 5 sought treatment guidelines (Table 4). In contrast, 3 out of 5 and 4 out of 5 of GPs aged 30–39 reported felt inadequately trained or expressed a need for treatment guidelines. Counts and percentages further revealed that GPs in Vienna were less likely to consider obesity a chronic disease (*p* = 0.036) and less familiar with multimodal therapy programs than those in Salzburg (*p* = 0.007, Table 5). Practice settings were associated with considering obesity a chronic disease (*p* = 0.005), referring patients to psychotherapists/psychologists (*p* = 0.017), perceiving barriers due to limited knowledge (*p* = 0.005), and the desire for disease management programs (*p* = 0.007). General practitioners in primary care units (PCUs) seemed more likely to reject obesity as a chronic condition. Conversely, GPs in solo and group practices seemed to perceive fewer barriers due to limited knowledge and less frequently requested disease management programs compared to those in PCUs and other settings (Table 5). GPs with private insurance contracts seemed to feel best trained to treat obesity (*p* = 0.023) and were less likely to refer patients to specialized obesity clinics (*p* = 0.022). They also showed the highest likelihood of offering long-term obesity treatment (*p* = 0.018) and were the only group that did not request additional support (*p* = 0.018, Table 5).

## Discussion

Our survey showed that participating GPs assessed themselves as responsible for the care of patients with overweight and obesity and considered the prevention and treatment of obesity a key primary care responsibility. This perceived responsibility aligns with the description of the role of GPs in providing comprehensive and continuous care according to the European guidelines for obesity management in primary care [1].

Despite a strong sense of responsibility, our survey showed that nearly half of the participants did not feel adequately trained to fulfill this task. This was also reflected by the fact that more than half of the participants have not conducted any or only a few long-term therapies so far. The feeling of inadequate education is particularly prevalent among less experienced and younger GPs, suggesting that obesity is not receiving enough attention in medical education and GP training. Data from the United States underline that the low priority given to obesity education in medical schools contributes to a lack of preparedness among medical students to effectively manage patients with obesity [18].

The majority of participating GPs consider obesity to be a chronic disease. This finding is broadly consistent with the results from other studies [17, 19]. Comparing the prevalence rates of obesity to other chronic diseases in Austria, it becomes clear that obesity is indeed one of the most common chronic diseases [20]. Despite the high disease burden, there is limited access to evidence-based therapeutic measures. Several drugs are now approved for obesity treatment and clearly recommended in the guidelines, but costs are generally not covered by health insurance. This fact was also identified by the participants in our study as a major problem for successful treatment.

Most respondents in our study would like a wider range of specialized resources for optimal patient care. As mentioned above, an interdisciplinary care approach is very important in treating obesity. However, in Austria, primary care still predominantly takes place in individual practices [8], which may hinder this approach, as dieticians, psychotherapists, or physiotherapists must be referred to externally. In addition, waiting times for insurance-covered therapy places are long, there are not enough places, or the services are not at all or not fully covered by health insurance.

Even if current services are assumed to be insufficiently available or widespread, it is crucial to note that existing services might not be well-known among GPs. In our survey, 44% of the respondents did not know any multimodal therapy program at all. Particularly in Vienna, such offerings are barely known to GPs. It is unclear whether this is due to differences in local healthcare infrastructure, training deficits, or a lower perceived priority of obesity in urban primary care. However, there is certainly a gap that needs to be closed, as many guidelines also clearly recommend referring patients to such a program when available [11, 13].

Structured DMPs, which were frequently requested by survey participants, could help address this issue by ensuring more structured and continuous care. In addition, DMPs can provide clear guidelines for the treatment of obesity. More than half of the survey respondents indicated that guidelines and educational opportunities are not sufficiently available. The Austrian Obesity Society (ÖAG) recently published a consensus paper with treatment recommendations for doctors of all specialties who treat people with obesity, thus, taking an important step forward [21].

Among barriers to optimal care, GPs in our study identified “lack of patient motivation” as the greatest challenge, a finding similar to that of other studies and surveys [5, 15, 16, 19]. This is neither a structural nor a GP-specific problem and could therefore be solved without a great expenditure of resources. Interestingly, GPs reported the lack of patient motivation across all demographic groups which is particularly concerning, as it is well known that the stigmatization of overweight people, both generally and in the medical context, is a serious problem and can significantly affect treatment outcomes [22–24]. Future efforts should focus on raising awareness and reducing biases among medical professionals. Current data indicate that around 60 to 70% of all overweight and/or obese people have already experienced weight stigmatization by physicians [23]. In addition, lack of time and inadequate reimbursement by health insurances were frequently mentioned as barriers. These challenges have a greater impact on the publicly funded system. Our results also showed that private doctors were most likely to provide long-term obesity treatment. Addressing disparities between the public and private sectors may be critical to ensuring equitable and effective obesity treatment in all practice settings.

When analyzing the results by gender, female physicians were more likely to screen for lifestyle factors such as dietary and physical activity habits, suggesting that they pay greater attention to prevention, an observation that is supported by existing literature [25]. However, they prescribed pharmacological treatments less frequently and referred patients to specialists more often. Additionally, female GPs expressed a greater demand for specific resources to support patients with obesity, such as specialized outpatient care and further training materials. This may reflect a different approach to obesity management according to gender, but might also indicate the well-known “gender gap”: women tend to underestimate their knowledge and skills, even though there are no objective differences to men [26]. These results are particularly noteworthy, given that there is a female majority in general medicine in Austria [9].

Furthermore, our results indicate that the practice setting plays an important role in the treatment of obesity. GPs in PCUs were less likely to consider obesity a chronic disease and reported limited knowledge about treatment options more frequently than GPs working in other settings. This difference raises the question of whether PCU structures need adjustments to better support obesity care. While patients may have broader access to medical and therapeutic services in PCUs, the continuity of care might on the other hand be reduced, as patients may see different physicians over time, potentially leading to a stronger focus on acute conditions rather than long-term management.

In summary, both structural changes and a greater awareness of the existing challenges in medical practice are needed to sustainably improve the care of people with obesity. The results of our study underscore the need for improved education and easier access to resources. Removing multiple barriers will be crucial to ensure that obesity is treated as the chronic disease that it is and that patients receive the best possible care.

## Strengths and limitations

This study has several limitations, and the results must be interpreted with caution. The number of participants is rather small, particularly in some demographic subgroups. The response rate is unknown due to the nature of data collection, which limits the ability to assess the representativeness of the findings. Additionally, selection bias may have influenced the study results, as GPs who participated were likely those with a particular interest in obesity management, potentially leading to an overestimation of awareness and engagement with obesity care. Another limitation is the reliance on self-assessment, which introduces a risk of response bias, as participants may overestimate or underestimate their knowledge and skills. Furthermore, the study’s exploratory nature means that it primarily serves as a foundation for future research rather than providing definitive conclusions. The use of a nonvalidated, self-developed questionnaire is another limitation, as it may not comprehensively capture all relevant aspects of obesity care and may lack reliability and validity, even though the questionnaire was pretested. Finally, we might not have collected all potentially relevant demographic variables, which could have provided further insights into potential influencing factors. To enhance the generalizability of findings, future studies should use larger sample sizes and a clearly defined conceptual model to enable regression analyses exploring the relationships between demographic variables and various aspects of screening and treatment. Additionally, employing validated survey instruments, expanding demographic data collection, and improving response assessment would enhance the generalizability of findings.

Nevertheless, the study provides a picture of the challenges GPs face in managing obesity and how they perceive these challenges in their daily practice. Despite its limitations, the findings align with results from other studies. This underlines the relevance of the topic and the need for further research and targeted interventions to improve obesity care in primary care settings.

## Supplementary Information

Questionnaire

Supplementary tables

## Abbreviations

DMP: Disease management program
GP: General practitioner
ÖAG: Österreichische Adipositas Gesellschaft
ÖGAM: Österreichische Gesellschaft für Allgemein- und Familienmedizin
PCU: Primary care unit
SAGAM: Salzburger Gesellschaft für Allgemein- und Familienmedizin
WIGAM: Wiener Gesellschaft für Allgemein- und Familienmedizin
