# Supplementary material for: Exploratory study on the self-perceived knowledge and care competence of general practitioners in managing patients with overweight and obesity in Austria
Source: Wien Klin Wochenschr. 2025 Jun 11;137(23-24):747–56. doi: 10.1007/s00508-025-02545-3 (PMC12712046; doi:10.1007/s00508-025-02545-3)
Supplement: Supplementary file 1 — Questionnaire [file 508_2025_2545_MOESM1_ESM.docx]

**Supplements**

**Questionnaire**

Personal Data – Question Group 1

1. Please indicate your gender:

- Male
- Female
- Diverse

1. How many years of professional experience do you have as a general practitioner?

- Less than 5 years
- 5-10 years
- 11-20 years
- More than 20 years

1. Please indicate your age:

- < 30 years
- 30-39 years
- 40-49 years
- 50-59 years
- 60-69 years
- 70 years

1. In which federal state are you practicing?

- Vienna
- Salzburg
- Other (please specify): _________

1. In what setting do you primarily practice general medicine?

- Solo practice
- Group practice
- Primary care unit
- Other (please specify): _________

1. Do you have a health insurance contract?

- Yes, with all insurance providers
- Yes, with specific insurance providers
- No, I work as a private or elective doctor
- No, I work as an employed doctor or as a substitute

Prevention and Screening – Question Group 2

1. To what extent do you agree with the following statement?: “The prevention of overweight and/or obesity is a family doctors task".

- Very strongly
- Strongly
- Barely
- Not at all

1. What parameters do you assess to screen for overweight and/or obesity and thereby identify potential risk groups? (Multiple answers possible)

- Body-Mass-Index (BMI)
- Waist-to-hip ratio
- Measurement of waist circumference
- Family history of overweight and/or obesity
- Dietary habits
- Activity level and physical exercise
- Blood tests (e.g., lipid profile, blood sugar)
- None
- Other

1. What measures do you implement in your practice to prevent overweight and/or obesity? (Multiple answers possible)

- Nutritional recommendations
- Physical activity recommendations
- Behavioral recommendations (e.g., topics like sleep hygiene and media consumption) Weight monitoring (e.g., annual assessment of weight/BMI)
- Pharmacological therapy
- Referral to specialists
- None
- Other

Therapy – Question Group 3

1. To what extent do you agree with the following statement?: “Obesity is a chronic disease.”

- Very strongly
- Strongly
- Barely
- Not at all

1. To what extent do you agree with the following statement?: “The therapy of overweight and/or obesity is a family doctors task".

- Very strongly
- Strongly
- Barely
- Not at all

1. To what extent do you agree with the following statement?: “I feel sufficiently trained to competently and professionally care for patients with overweight and/or obesity."

- Very strongly
- Strongly
- Barely
- Not at all

1. To what extent do you agree with the following statement?: "As a general practitioner, I see myself as the primary physician responsible for the care of my patients with overweight and/or obesity."

- Very strongly
- Strongly
- Barely
- Not at all

1. With how many of your overweight and/or obese patients do you discuss their weight? Please provide the value as a percentage:"
2. What measures do you implement in your practice to treat overweight and/or obesity? (Multiple answers possible)

- Nutritional recommendations
- Physical activity recommendations
- Behavioral recommendations (e.g., topics like sleep hygiene and media consumption) Weight monitoring (e.g., annual assessment of weight/BMI)
- Pharmacological therapy
- Referral to specialists
- None
- Other

1. To which healthcare providers or care facilities do you refer patients with overweight and/or obesity?

- Specialists in outpatient practice
- Dietitians/Nutritionists
- Physiotherapists/Exercise therapists
- Psychotherapists/Psychologists
- Obesity clinics/Obesity centers
- None
- Other

1. “Have you already conducted long-term treatments for patients with overweight and/or obesity in your practice?“ *This refers to continuous and structured follow-up care beyond an initial consultation.*

- Yes, very many
- Yes, many
- Yes, a few
- No, none

8a. *This question was only shown to the 47 individuals who answered question 8 with 'Yes,...':* Which patient group(s) have you treated so far?

- Children and adolescents (up to 17 years)
- Adults (18-64 years)
- Older patients (over 65 years)

1. Are you familiar with specific multimodal therapy programs for the treatment of overweight and/or obesity? If yes, please provide the names of the programs (Multiple answers possible).

- Yes, I am familiar with such
- No, I am not familiar with any such programs

9a. *This question was only shown to the 33 individuals who answered question 9 with 'Yes, ...':* „Have you already referred patients to multimodal therapy programs?“

- Yes, very many
- Yes, many
- Yes, a few
- No, none

1. "What barriers do you experience in providing optimal care for patients with overweight and/or obesity?” (Multiple answers possible)

- Lack of patient motivation
- Lack of time for thorough counseling
- Limited knowledge about current treatment options
- Lack of reimbursement for medical services by health insurance
- Costs incurred by patients
- None
- Other

1. "What support would you like to improve the care of patients with overweight and/or obesity?” (Multiple answers possible)

- Training programs on current treatment options
- Broader availability of specialized resources for patients (e.g., multimodal therapy programs, obesity centers)
- Improved interdisciplinary collaboration (e.g., with specialists, dietitians, psychotherapists, physiotherapists)
- Guidelines and recommendations for the treatment of overweight and obesity
- Disease management programs (e.g., Therapy Active for Diabetes)
- None
- Other
