# Supplementary material for: Exploratory study on the self-perceived knowledge and care competence of general practitioners in managing patients with overweight and obesity in Austria
Source: Wien Klin Wochenschr. 2025 Jun 11;137(23-24):747–56. doi: 10.1007/s00508-025-02545-3 (PMC12712046; doi:10.1007/s00508-025-02545-3)
Supplement: Supplementary file 2 — Supplementary tables [file 508_2025_2545_MOESM2_ESM.docx]

**Supplementary Tables**

Supplementary Table 1: Results on questions about screening and prevention of overweight and obesity

| **1. To what extent do you agree with the following statement? “The prevention of overweight and/or obesity is a family doctors responsibility".** | | | |
| --- | --- | --- | --- |
|  | **N=59** | **Percentage** | |
| Very strongly | 24 | 41% | |
| Strongly | 28 | 47% | |
| Barely | 7 | 12% | |
| Not at all | 0 | 0% | |
|  | | | |
| **2. What parameters do you assess to screen for overweight and/or obesity and thereby identify potential risk groups?** (Multiple answers possible) | | | |
|  | | | **Percentage** |
| Body-Mass-Index (BMI) | | | 97% |
| Waist-to-hip ratio | | | 17% |
| Measurement of waist circumference | | | 59% |
| Family history of overweight and/or obesity | | | 59% |
| Dietary habits | | | 75% |
| Activity level and physical exercise | | | 80% |
| Blood tests (e.g., lipid profile, blood sugar) | | | 83% |
| None | | | 0% |
| Other | | | 5% |
|  | | | |
| **3. What measures do you implement in your practice to prevent overweight and/or obesity?** (Multiple answers possible) | | | |
| **Antwort** | | **Percentage** | |
| Nutritional recommendations | | 93% | |
| Physical activity recommendations | | 100% | |
| Behavioral recommendations (e.g., topics like sleep hygiene and media consumption) | | 51% | |
| Weight monitoring (e.g., annual assessment of weight/BMI) | | 59% | |
| Pharmacological therapy | | 17% | |
| Referral to specialists | | 49% | |
| None | | 0% | |
| Other | | 3% | |

For questions with multiple answer options, only the percentage values are displayed in the tables for clarity.

Supplementary Table 2: Results on questions about treatment of overweight and obesity

| **1. To what extent do you agree with the following statement? “Obesity is a chronic disease.”** | | | |
| --- | --- | --- | --- |
|  | **N=59** | **Percentage** | |
| Very strongly | 41 | 70% | |
| Strongly | 15 | 25% | |
| Barely | 3 | 5% | |
| Not at all | 0 | 0% | |
|  | | | |
| **2. To what extent do you agree with the following statement? “The therapy of overweight and/or obesity is a family doctors responsibility".** | | | |
|  | **N=59** | **Percentage** | |
| Very strongly | 20 | 34% | |
| Strongly | 34 | 58% | |
| Barely | 5 | 8% | |
| Not at all | 0 | 0% | |
|  | | | |
| **3. To what extent do you agree with the following statement? “I feel sufficiently trained to competently and professionally care for patients with overweight and/or obesity."** | | | |
|  | **N=59** | **Percentage** | |
| Very strongly | 8 | 14% | |
| Strongly | 23 | 39% | |
| Barely | 28 | 47% | |
| Not at all | 0 | 0% | |
|  | | | |
| **4. To what extent do you agree with the following statement? "As a general practitioner, I see myself as the primary physician responsible for the care of my patients with overweight and/or obesity."** | | | |
|  | **N=59** | **Percentage** | |
| Very strongly | 17 | 29% | |
| Strongly | 37 | 63% | |
| Barely | 5 | 8% | |
| Not at all | 0 | 0% | |
|  | | | |
| **5. With how many of your overweight and/or obese patients do you discuss their weight? Please provide the value as a percentage:"** | | | |
|  | **Mean** | **Median** | |
|  | 65% | 75% | |
|  | | | |
| **6. What measures do you implement in your practice to treat overweight and/or obesity?** (Multiple answers possible) | | | |
|  | | | **Percentage** |
| Nutritional recommendations | | | 100% |
| Physical activity recommendations | | | 95% |
| Behavioral recommendations (e.g., topics like sleep hygiene and media consumption) | | | 54% |
| Pharmacological therapy | | | 47% |
| None | | | 0% |
| Other | | | 15% |
|  | | | |
| **7. To which healthcare providers or care facilities do you refer patients with overweight and/or obesity? (**Multiple answers possible) | | | |
|  | | | **Percentage** |
| Specialists in outpatient practice | | | 42% |
| Dietitians/Nutritionists | | | 85% |
| Physiotherapists/Exercise therapists | | | 39% |
| Psychotherapists/Psychologists | | | 46% |
| Obesity clinics/Obesity centers | | | 76% |
| None | | | 2% |
| Other | | | 8% |
|  | | | |
| **8. “Have you already conducted long-term treatments for patients with overweight and/or obesity in your practice? “** *This refers to continuous and structured follow-up care beyond an initial consultation.* | | | |
|  | **N=59** | **Percentage** | |
| Yes, very many | 6 | 10% | |
| Yes, many | 18 | 31% | |
| Yes, a few | 23 | 39% | |
| No, none | 12 | 20% | |
|  | | | |
| **8a.** *This question was only shown to the 47 individuals who answered question 8 with 'Yes,...':* **Which patient group(s) have you treated so far?** (Multiple answers possible) | | | |
|  | | **Percentage** | |
| Children and adolescents (up to 17 years) | | 32% | |
| Adults (18-64 years) | | 100% | |
| Older patients (over 65 years) | | 45% | |
|  | | | |
| **9. Are you familiar with specific multimodal therapy programs for the treatment of overweight and/or obesity? If yes, please provide the names of the programs** (Multiple answers possible)**.** | | | |
|  | **N=59** | **Percentage** | |
| Yes, I am familiar with such programs   - Leichter Leben / easykids / enorm in Form / AVOS-Programme - Optifast / Adipomed - Leicht durchs Leben - Coping School BHS Wien - Anderes | 33   - 15 - 13 - 1 - 2 - 11 | 56% | |
| No, I am not familiar with any such programs. | 26 | 44% | |
|  | | | |
| **9a.** *This question was only shown to the 33 individuals who answered question 9 with 'Yes, ...':* **„Have you already referred patients to multimodal therapy programs? “** | | | |
|  | **N=33** | **Percentage** | |
| Yes, very many | 0 | 0% | |
| Yes, many | 6 | 18% | |
| Yes, a few | 24 | 73% | |
| No, none | 3 | 9% | |
|  | | | |
| **10. "What barriers do you experience in providing optimal care for patients with overweight and/or obesity?”** (Multiple answers possible) | | | |
|  | | | **Percentage** |
| Lack of patient motivation | | | 81% |
| Lack of time for thorough counseling | | | 71% |
| Limited knowledge about current treatment options | | | 29% |
| Lack of reimbursement for medical services by health insurance | | | 75% |
| Costs incurred by patients | | | 71% |
| None | | | 0% |
| Other | | | 19% |
|  | | | |
| **11. "What support would you like to improve the care of patients with overweight and/or obesity?”** (Multiple answers possible) | | | |
|  | | | **Percentage** |
| Training programs on current treatment options | | | 53% |
| Broader availability of specialized resources for patients (e.g., multimodal therapy programs, obesity centers) | | | 81% |
| Improved interdisciplinary collaboration (e.g., with specialists, dietitians, psychotherapists, physiotherapists) | | | 64% |
| Guidelines and recommendations for the treatment of overweight and obesity | | | 56% |
| Disease management programs (e.g., Therapy Active for Diabetes) | | | 69% |
| None | | | 0% |
| Other | | | 8% |
|  | | | |

For questions with multiple answer options, only the percentage values are displayed in the tables for clarity.
